# FMDV 3A Antagonizes the Effect of ANXA1 to Positively Modulate Viral Replication

**DOI:** 10.1128/jvi.00317-22

**Published:** 2022-05-23

**Authors:** XuSheng Ma, KeShan Zhang, ZhiKuan Luo, XiaoFeng Nian, S. K. Mohiuddin Choudhury, ZiXiang Zhu, Rui Song, JingJing Pei, YanLi Huo, YuanYuan Li, Fan Yang, WeiJun Cao, HuiSheng Liu, XiangTao Liu, HaiXue Zheng

**Affiliations:** a State Key Laboratory of Veterinary Etiological Biology, National Foot and Mouth Diseases Reference Laboratory, Key Laboratory of Animal Virology of Ministry of Agriculture, Lanzhou Veterinary Research Institute, Chinese Academy of Agricultural Sciences, Lanzhou, Gansu, China; b School of Chemical and Biological Engineering, Lanzhou Jiaotong University, Lanzhou, China; c Gansu Agricultural University, Lanzhou, China; Instituto de Biotecnologia/UNAM

**Keywords:** 3A, ANXA1, foot-and-mouth disease virus, interferons, viral replication

## Abstract

The RIG-I-like receptor signaling pathway is crucial for producing type I interferon (IFN-I) against RNA viruses. The present study observed that viral infection increased annexin-A1 (ANXA1) expression, and ANXA1 then promoted RNA virus-induced IFN-I production. Compared to ANXA1 wild-type cells, ANXA1^−/−^ knockout cells showed IFN-β production decreasing after viral stimulation. RNA virus stimulation induced ANXA1 to regulate IFN-β production through the TBK1-IRF3 axis but not through the NF-κB axis. ANXA1 also interacted with JAK1 and STAT1 to increase signal transduction induced by IFN-β or IFN-γ. We assessed the effect of ANXA1 on the replication of foot-and-mouth disease virus (FMDV) and found that ANXA1 inhibits FMDV replication dependent on IFN-I production. FMDV 3A plays critical roles in viral replication and host range. The results showed that FMDV 3A interacts with ANXA1 to inhibit its ability to promote IFN-β production. We also demonstrated that FMDV 3A inhibits the formation of ANXA1-TBK1 complex. These results indicate that ANXA1 positively regulates RNA virus-stimulated IFN-β production and FMDV 3A antagonizes ANXA1-promoted IFN-β production to modulate viral replication.

**IMPORTANCE** FMDV is a pathogen that causes one of the world’s most destructive and highly contagious animal diseases. The FMDV 3A protein plays a critical role in viral replication and host range. Although 3A is one of the viral proteins that influences FMDV virulence, its underlying mechanisms remain unclear. ANXA1 is involved in immune activation against pathogens. The present study demonstrated that FMDV increases ANXA1 expression, while ANXA1 inhibits FMDV replication. The results also showed that ANXA1 promotes RNA virus-induced IFN-I production through the IRF3 axis at VISA and TBK1 levels. ANXA1 was also found to interact with JAK1 and STAT1 to strengthen signal transduction induced by IFN-β and IFN-γ. 3A interacted with ANXA1 to inhibit ANXA1-TBK1 complex formation, thereby antagonizing the inhibitory effect of ANXA1 on FMDV replication. This study helps to elucidate the mechanism underlying the effect of the 3A protein on FMDV replication.

## INTRODUCTION

Foot-and-mouth disease virus (FMDV) belongs to the *Picornaviridae* family and is a pathogen that causes one of the world’s most destructive and highly contagious animal diseases. The FMDV genome encodes 12 proteins, including four structural proteins (VP1 to -4) and eight nonstructural proteins (L^pro^, 2A, 2B, 2C, 3A, 3B, 3C, and 3D) (1). The FMDV nonstructural proteins together with some host proteins constitute viral replication sites. The 3A protein, through its hydrophobic motif, anchors on the intracellular membrane of the host cell (2). This protein is an essential component for FMDV replication complex formation. It mediates the localization of the viral replication complex on cell membrane structure. Although FMDV 3A is one of the viral proteins that influence FMDV virulence, its underlying mechanisms remain unclear (3–5).

The RIG-I like receptors (RLRs) pathway is the most prominent innate immune signaling pathway for sensing cytosolic RNA. The RIG-I or MDA5 signaling pathway induces NF-κB- or IRF3-mediated IFN-I production after viral stimulation (6). Type I interferon (IFN-I) interacts with the receptors and activates JAK1 and Tyk2 function, which phosphorylates STAT2 and STAT1 to translocate into the nucleus, thereby leading to antiviral IFN-stimulated gene expression (7). The host factors significantly regulate RLR signaling. For instance, TRIM25 induces the degradation of VISA to activate the signaling transduction of the IFN-I pathway through the K48-linked ubiquitination at Lys7 and Lys10 (8); the scaffold protein FAS-associated factor 1 (FAF1) disrupts the interactions of VISA and TRIM31 to negatively regulate TRIM31-mediated ubiquitination of VISA (9). Here, we found that annexin-A1 (ANXA1) can regulate RNA virus-induced RLR signaling.

ANXA1 is a member of the annexin superfamily, and it is widely expressed in many tissues, including immune and epithelial cells. Previous studies have shown that ANXA1 is involved in immune activation against pathogens. ANXA1 interacts with FPR2 to enhance the functionality of alveolar macrophages and inhibits murine influenza A virus infection (10, 11). In the absence of ANXA1, the response to TLR4-induced activation is reduced in bone marrow-derived cells (BMDCs) (12). ANXA1 promotes TLR3- and TLR4-induced IFN-β production to influence the innate immune response (13). ANXA1 activates NF-κB through its interaction with NEMO and RIP1 in breast cancer cells (14). Recently, Yap et al. reported that ANXA1 enhances cell apoptosis after influenza type A virus (IAV) infection (15). ANXA1 increases RIG-I activation, which induces apoptosis of A549 lung epithelial cell by regulating the IRF3-IFNAR-STAT1-IFIT1 pathway (15). Although ANXA1 is involved in innate immune responses, the mechanism of regulation of FMDV-induced IFN-β production is not entirely understood.

In the present study, we found that viral infection increased ANXA1 expression. Moreover, ANXA1 overexpression facilitated the RNA virus-induced antiviral response, while ANXA1 knockout decreased virus-induced IFN-I production and antiviral gene expression. ANXA1 interacted with VISA and TBK1 to regulate RNA virus-induced IFN-β production through the TBK1-IRF3 axis rather than through the NF-κB axis. ANXA1 also interacted with JAK1 and STAT1 to increase signal transduction induced by IFN-β and IFN-γ. We demonstrated that the FMDV 3A protein interacts with ANXA1 to antagonize ANXA1-mediated promotion of virus-induced IFN-β production and to counteract ANXA1-mediated inhibition of FMDV replication. The results also showed that 3A inhibits ANXA1-TBK1 complex formation. Our results indicated that ANXA1 positively regulates RNA virus-stimulated IFN-β production and IFN-β/γ-induced JAK-STAT signal transduction. However, FMDV 3A disrupts ANXA1-TBK1 complex formation to block IFN-β production and thus promotes FMDV replication.

## RESULTS

### ANXA1 increased after viral infection or IFN-β stimulation.

**FIG 1:**
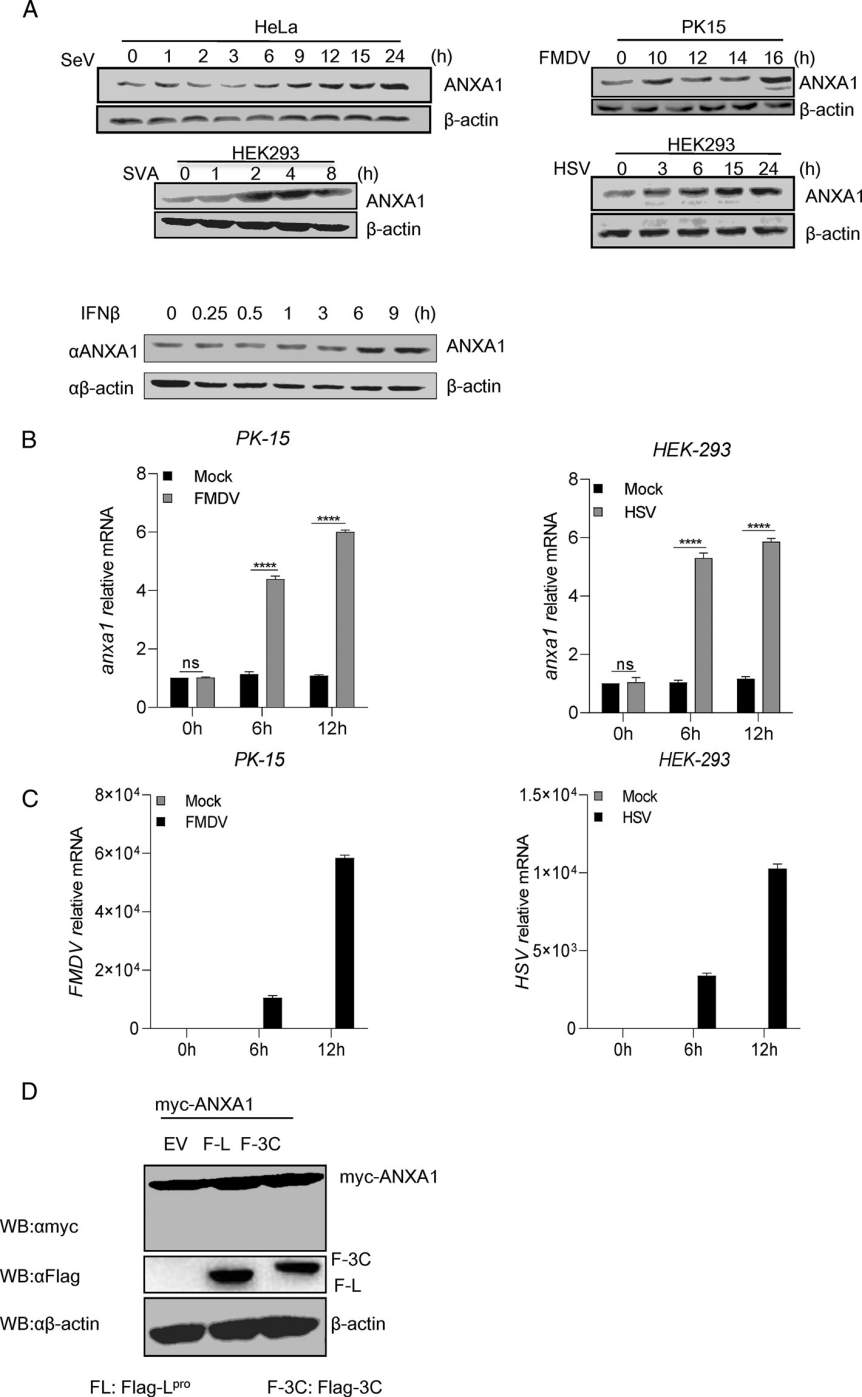
Viral infection and IFN-β stimulation increased ANXA1 expression. (A) HeLa, PK15, or HEK293 cells were infected with SeV, FMDV, SVA, or HSV (MOI of 4) at the indicated time points. HEK293 cells were stimulated with IFN-β at the indicated time points. Cell lysates were subjected to Western blotting (WB). (B and C) PK15 or HEK293 cells were infected with FMDV and HSV (MOI of 4) at the indicated time points. Cellular RNA was extracted and reverse transcribed into cDNA. Compared to the control, relative mRNA levels of ANXA1 (B), FMDV, and HSV (C) were detected by qPCR. (D) Plasmids of EV (empty vector), FMDV 3C (F-3C, Flag-3C), or FMDV L (FL, Flag-L^pro^) together with ANXA1 were transfected into HEK293T cells separately. After 24 h, cell lysates were subjected to Western blotting. Data are representative of three independent experiments. The data are expressed as means ± SEM; *, *P < *0.05; **, *P < *0.01; ***, *P < *0.001; ****, *P < *0.0001 (two-way ANOVA; GraphPad Prism 8.3.0).

To verify whether the expression level of ANXA1 is correlated with viral infection, we assessed ANXA1 protein expression and mRNA levels after viral infection. As shown in Fig. 1A, ANXA1 protein expression was increased after PK-15 cells, HeLa cells, or HEK293 cells were infected with FMDV, Sendai virus (SeV), Seneca valley virus (SVA), or herpes simplex virus (HSV). Interestingly, ANXA1 showed two bands after FMDV infection at 16 h. Previous study showed that ANXA1 increased IFN-I production through Toll-like receptors (TLRs) (13). To determine the effect of IFN-β on ANXA1 expression, we detected ANXA1 protein expression after IFN-β treatment at indicated time points. We found that IFN-β also increased ANXA1 protein expression (Fig. 1A). As shown in Fig. 1B, we verified that the mRNA levels of ANXA1 were increased in FMDV-infected PK-15 cells and HSV-infected HEK293 cells compared to that in the untreated control cells. Consistent with this, the mRNA expression of FMDV and HSV also increased at 6 and 12 h postinfection (Fig. 1C). FMDV 3C and L protease can cleave host proteins (14, 16). Thus, to determine whether FMDV 3C and L protease play a role in ANXA1 cleavage, ANXA1 together with 3C or L^pro^ were transfected into HEK293 cells. As shown in Fig. 1D, FMDV 3C and L^pro^ did not affect ANXA1 expression level.

### ANXA1 enhances SeV-induced IFN-I activation.

**FIG 2:**
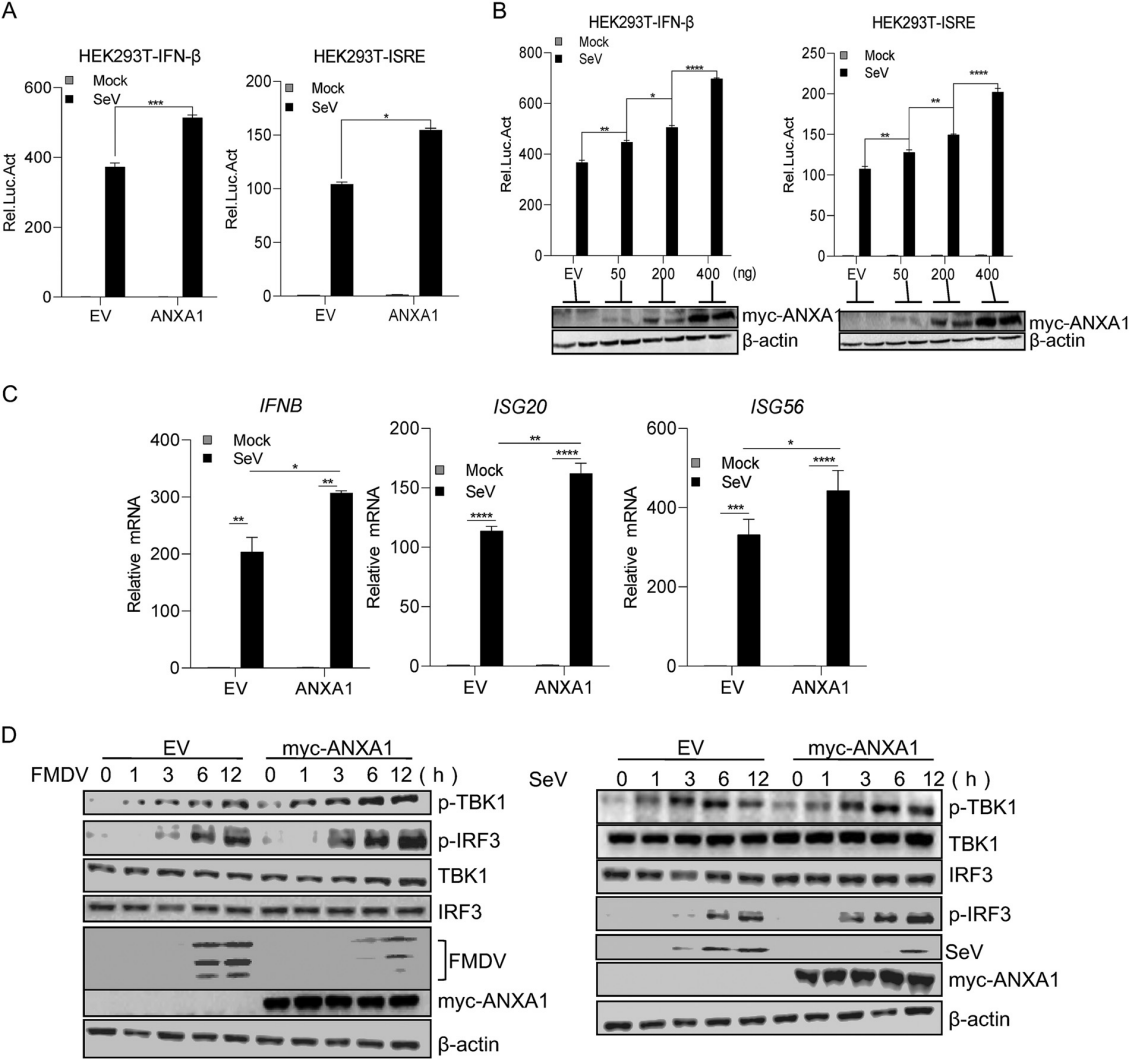
ANXA1 enhanced SeV-induced IFN-I activation. (A) Plasmids of ANXA1 (300 ng) and pRL-TK (20 ng) together with pIFN-β-luc or pISRE-luc (200 ng) were transfected into HEK293T cells. After 24 h, the cells were infected with SeV, and after 12 h, the cell lysates were detected with the relative intensity of luciferase. (B) Plasmids containing ANXA1 (indicated dose) and pRL-TK (20 ng) together with pIFN-β-luc or pISRE-luc (200 ng) were transfected into HEK293T cells. After 24 h, the cells were infected with SeV, and after 12 h, the cell lysates were detected with the relative intensity of luciferase. (C) ANXA1 plasmid (1 μg) was transfected into HEK293T cells. After 24 h, the cells were infected with SeV. After 12 h, cellular RNA was extracted and reverse transcribed into cDNA. Relative mRNA levels were detected by qPCR. (D) ANXA1 (2 μg) was transfected into PK15 or HEK293T cells. After 24 h, the cells were infected with FMDV or SeV at the indicated time points and cell lysates were subjected to Western blotting. Data are representative of three independent experiments. The data are expressed as means ± SEM; *, *P < *0.05; **, *P < *0.01; ***, *P < *0.001; ****, *P < *0.0001 (two-way ANOVA; GraphPad Prism 8.3.0).

**FIG 3:**
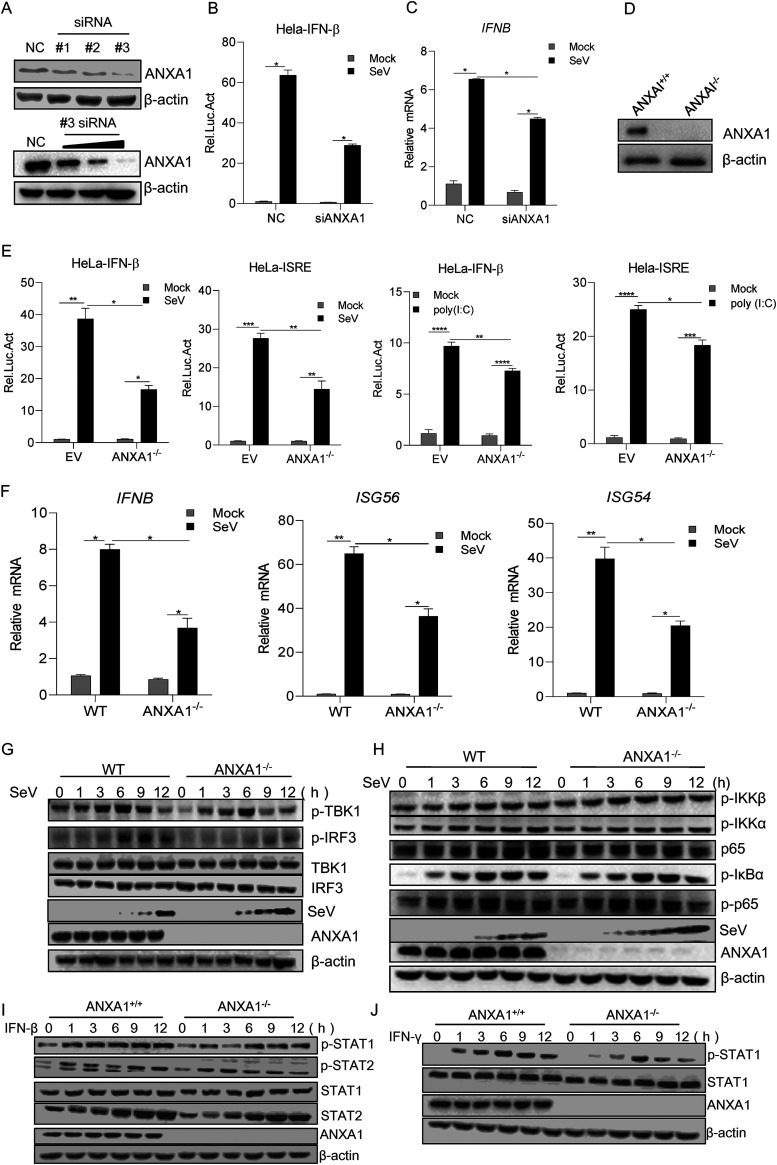
ANXA1 deficiency decreased virus-induced IFN-I activation. (A) ANXA1 siRNAs or negative ANXA1 siRNA control (NC) were transfected into HEK293T cells, and after 72 h, the cell lysates were subjected to Western blotting. Negative ANXA1 siRNA control (NC) or different doses of #3 ANXA1 siRNA were transfected into HEK293T cells, and after 72 h, the cell lysates were subjected to Western blotting. (B) Negative ANXA1 siRNA control (NC) or #3 ANXA1 siRNA was transfected into HeLa cells. After 48 h, the cells were transfected with IFN-β-luc, and at 24 h posttransfection, the cells were infected with SeV for 12 h; luciferase reporter gene activity was then detected. (C) ANXA1 siRNAs or negative ANXA1 siRNA control (NC) was transfected into HeLa cells, and after 72 h, the cells were infected with SeV for 12 h. Cellular RNA was extracted and reverse transcribed into cDNA. The relative mRNA level was detected by qPCR. (D) ANXA1 wild-type and ANXA1 knockout HeLa cell lysates were subjected to Western blotting. (E) ANXA1-knockout cells were transfected with pRL-TK (20 ng) together with pIFN-β-luc or pISRE-luc (400 ng). After 24 h, the cells were infected with SeV for 12 h or transfected with poly(I:C) (1 μg) for 24 h, and the cell lysates were detected with the relative intensity of luciferase. (F) ANXA1-knockout cells were infected with SeV, and after 12 h, cellular RNA was extracted and reverse transcribed into cDNA. The relative mRNA levels were detected by qPCR. (G and H) ANXA1-knockout cells or ANXA1 wild-type cells were infected with SeV at the indicated time points, and cell lysates were subjected to Western blotting. (I to J) ANXA1-knockout cells or ANXA1 wild-type cells were stimulated with IFN-β or IFN-γ at the indicated time points, and cell lysates were subjected to Western blotting. Data are representative of three independent experiments. The data are expressed as means ± SEM; *, *P < *0.05; **, *P < *0.01; ***, *P < *0.001; ****, *P < *0.0001 (two-way ANOVA; GraphPad Prism 8.3.0).

To evaluate whether ANXA1 regulates RNA virus-induced IFN-I induction, we detected SeV-induced IFN-β promoter activation in ANXA1-overexpressing HEK293T cells by using a luciferase reporter assay. The results revealed that ANXA1 promotes SeV-induced IFN-β and IFN-sensitive response element (ISRE) activation (Fig. 2A), suggesting that ANXA1 positively regulates virus-induced IFN-β production. As shown in Fig. 2B, ANXA1 enables IFN-β and ISRE activation in a dose-dependent manner. Interferon-stimulated genes (ISGs) are numerous antiviral factors induced by IFNs. The expression of *ISG20* and *ISG56* is induced after viral infection, and both these genes have the function of inhibition of viral replication (17–19). To confirm the accuracy of the effect of ANXA1 on RNA virus-induced IFN-β production, the mRNA levels of ISGs were detected in ANXA1-overexpressing HEK293T cells infected with SeV. The results showed that the mRNA levels of *IFNB*, *ISG20*, and *ISG56* were increased in ANXA1-overexpressing cells after SeV infection (Fig. 3C). To validate the effect of ANXA1 on IFN-I production signaling, ANXA1 was transfected into PK15 or HEK293 cells, and the cells were then infected with FMDV or SeV. The phosphorylation levels of TBK1 and IRF3 were then detected. As shown in Fig. 2D, ANXA1 increased the phosphorylation levels of IRF3 and TBK1 after FMDV or SeV infection. These results suggest that ANXA1 promotes RNA virus-induced IFN-β production.

### ANXA1 deficiency impairs RNA virus-induced IFN-I production.

To determine the effect of endogenous ANXA1 on SeV-induced innate immune response, the ANXA1 short interfering RNA (siRNA) was used to block endogenous ANXA1 protein expression. As shown in Fig. 3A, the modulation of #3 siRNA against ANXA1 was more significant, and it was further used for inhibition study. The role of ANXA1 has been extensively investigated in cancer cells. In our study, HeLa cells were used as the ANXA1 knockdown or knockout cell model (ANXA1^−/−^) to clarify how ANXA1 modulates the innate immune system. After the treatment of HeLa cells with ANXA1 siRNA, IFN-β promoter activation (Fig. 3B) and *IFNB* transcription (Fig. 3C) were observed to be decreased during SeV infection. We also found that IFN-β and ISRE promoter activation was reduced in ANXA1^−/−^ cells compared to that in wild-type (ANXA1^+/+^) HeLa cells after SeV or poly(I·C) treatment (Fig. 3D and E). Additionally, we found that the mRNA expression levels of ISGs, including *IFNB*, *ISG56*, and *ISG54*, were reduced after SeV infection in ANXA1^−/−^ cells (Fig. 3F). The role of ANXA1 in IFN-I signaling was assessed, and we found that TBK1 and IRF3 phosphorylation (but not p65 phosphorylation) was attenuated in SeV-infected ANXA1^−/−^ cells compared to that in ANXA1^+/+^ cells (Fig. 3G and H).

IFN-β secretion induced phosphorylation of STAT1 and STAT2 proteins. However, IFN-γ induced phosphorylation of only STAT1. To determine the effect of ANXA1 on IFN-induced signaling transduction, the phosphorylation of STATs was assessed. As shown in Fig. 3I, STAT1 phosphorylation decreased after IFN-β stimulation in ANXA1^−/−^ cells. Figure 3J shows that upon IFN-γ stimulation, the phosphorylation of STAT1 was reduced in ANXA1^−/−^ cells. Collectively, ANXA1 promoted RNA virus-induced IFN-β production and IFN-β/γ stimulated JAK-STAT signaling transduction.

**FIG 4:**
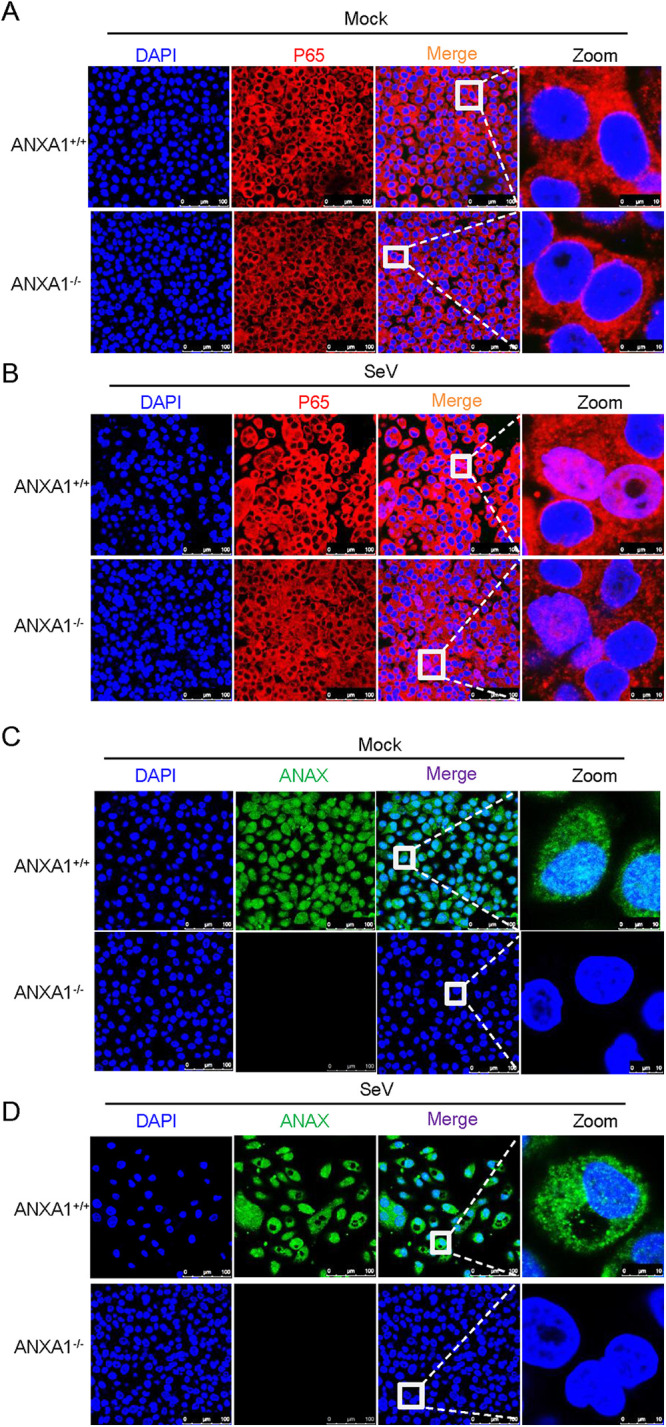
ANXA1 does not affect p65 distribution. (A to D) ANXA1^+/+^ or ANXA1^−/−^ cells were treated with SeV or left untreated. After 12 h, the cells were fixed and subjected to confocal imaging to detect the p65 and ANXA1 distribution. Data are representative of three independent experiments. The data are expressed as means ± SEM; *, *P < *0.05; **, *P < *0.01; ***, *P < *0.001; ****, *P < *0.0001 (two-way ANOVA; GraphPad Prism 8.3.0).

Next, to determine the effect of ANXA1 on NF-κB signaling, the localization of p65 in ANXA1^−/−^ cells was detected. We found that p65 localization did not change in noninfected ANXA1^−/−^ cells. Moreover, no significant difference was observed in the proportion of the p65 protein transported to the nucleus between ANXA1 wild-type and ANXA1^−/−^ cells after SeV infection (Fig. 4A to D). These results suggest that ANXA1 promotes SeV-induced IFN-β production through the TBK1-IRF3 axis.

### ANXA1 interacts with VISA and TBK1.

**FIG 5:**
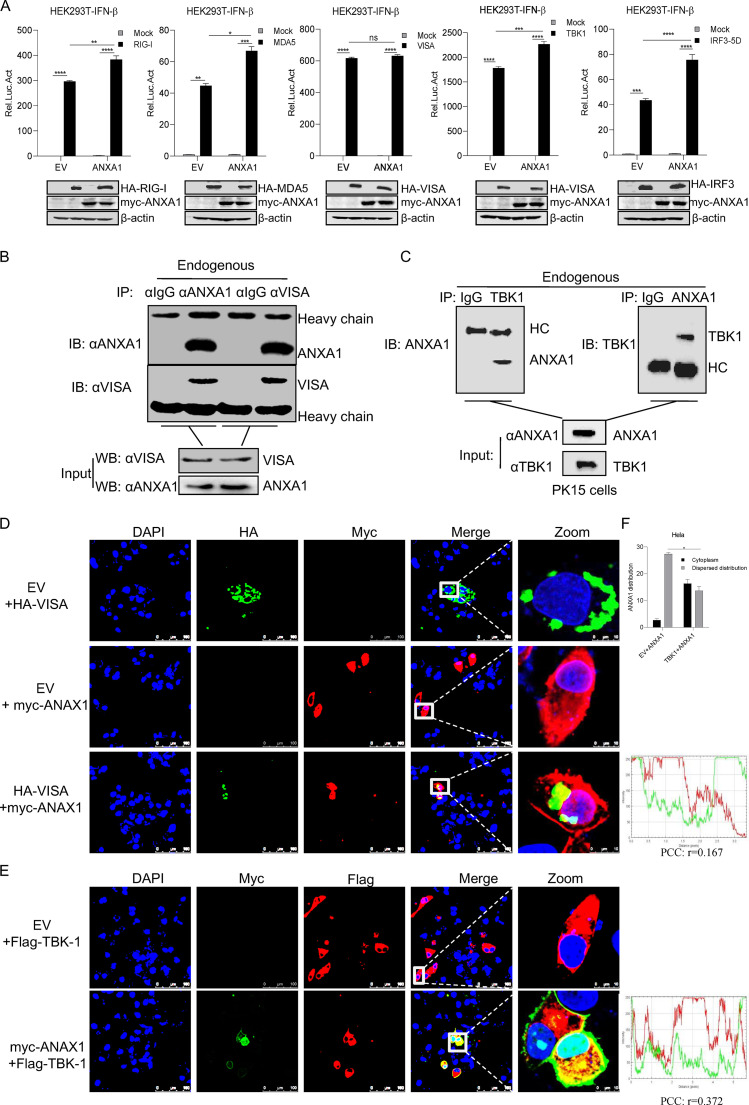
ANXA1 targeted VISA and TBK1 to affect IFN-β production. (A) Plasmids of ANXA1 (500 ng), pIFN-β-luc (200 ng), and pRL-TK (20 ng) together with RIG-I, MDA5, VISA, TBK1, or IRF3-5D (500 ng) were transfected into HEK293T cells, and after 24 h, the cell lysates were detected with the relative intensity of luciferase. (B) HeLa cells were cultured for 24 h, and cell lysates were subjected to co-IP. Anti-ANXA1 or anti-VISA antibodies were used as IP primary antibodies. Anti-ANXA1 and anti-VISA antibodies were used for IB to detect ANXA1 and VISA interaction. (C) HeLa cell lysates were subjected to co-IP using the indicated antibodies. Anti-TBK1 or anti-ANXA1 antibodies were used as IP primary antibodies. (D) HA-VISA together with EV or myc-ANXA1 were transfected into HeLa cells, and after 24 h, the cells were fixed and subjected to confocal imaging. (E) EV or myc-ANXA1 together with Flag-TBK1 was transfected into HeLa cells, and after 24 h, the cells were fixed and subjected to confocal imaging. (F) The distribution of ANXA1 in cells was analyzed. Thirty cells were observed, and the number of cells that ANXA1 dispersed into cytoplasm and nucleus and the number of cells that ANXA1 distributed around the membrane were statistically analyzed. Data are representative of three independent experiments. The data are expressed as means ± SEM; *, *P < *0.05; **, *P < *0.01; ***, *P < *0.001; ****, *P < *0.0001 (two-way ANOVA; GraphPad Prism 8.3.0).

Next, to investigate which components of the RLR signaling pathway are involved in ANXA1 regulation, MDA5, RIG-I, VISA, TBK1, or IRF3 was cotransfected with ANXA1 into HEK293T cells. As shown in Fig. 5A, we found that ANXA1 expression significantly promoted RIG-I-, MDA5-, TBK1-, and IRF3-5D (a constitutive active mutant form of IRF3)-mediated IFN-β production. This result suggests that ANXA1 expression affects IFN-β production at the VISA or IRF3 level. Next, to determine whether ANXA1 interacts with VISA, IRF3, or IRF3-related protein, ANXA1 was cotransfected with VISA, TBK1, or IRF3 into HEK293T cells. The results of coimmunoprecipitation (co-IP) assay showed that ANXA1 interacts with VISA (Fig. 5B) and TBK1 (Fig. 5C) but not with IRF3 (data not shown). In addition, in the fluorescence assay, both VISA and TBK1 were found to be colocalized with ANXA1 in cytoplasm (Fig. 5D and E), and Pearson's correlation coefficient (PCC) also confirmed these results. We also found that ANXA1 was distributed in the cytoplasm and nucleus, but it was localized only in the cytoplasm after TBK1 overexpression (Fig. 5F). These results indicated that ANXA1 interacts with VISA and TBK1 to promote virus-induced IFN-I production.

### ANXA1 interacts with JAK1 and STAT1.

**FIG 6:**
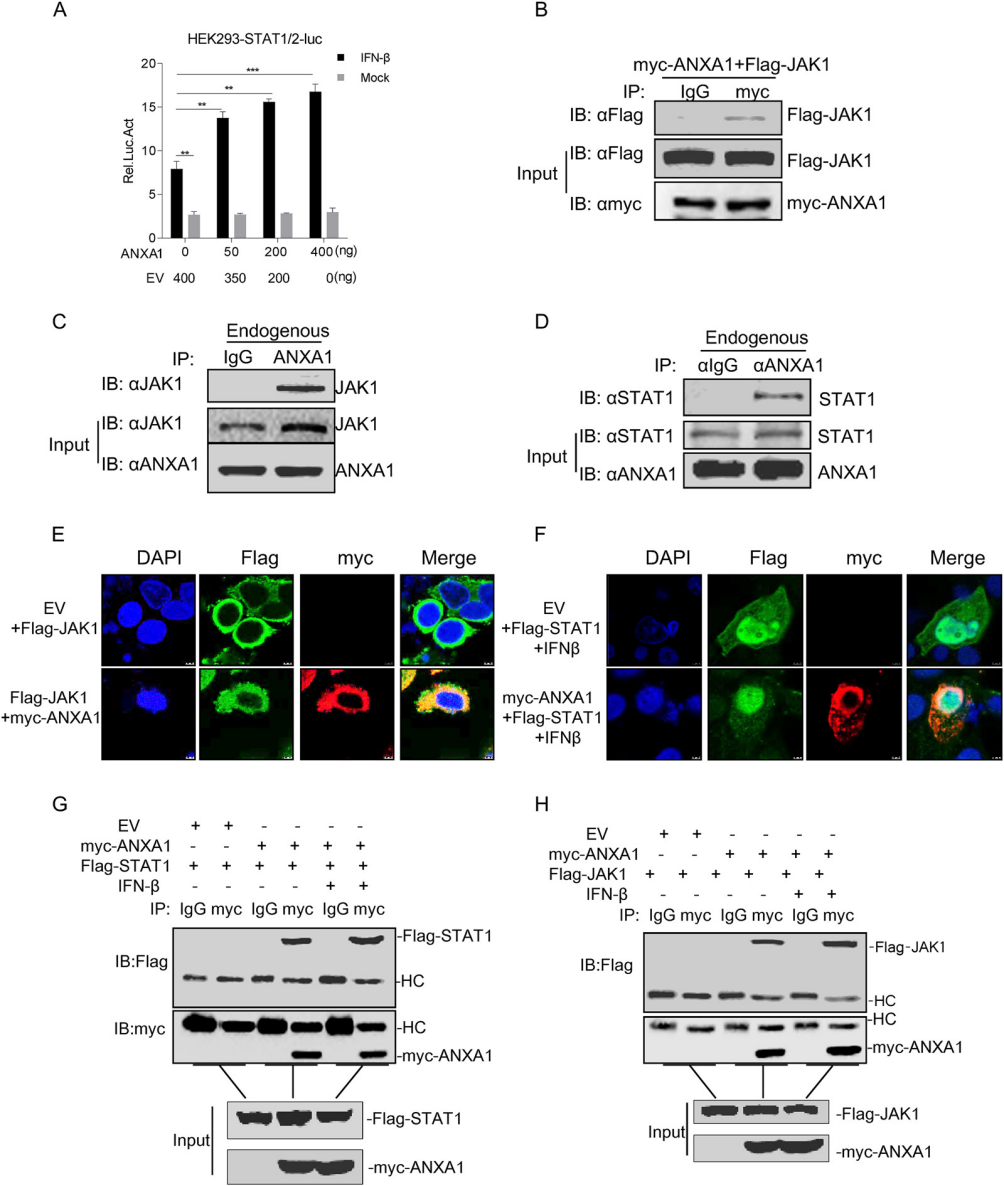
IFN-β stimulation has no effect on the interaction between ANXA1 and STAT1 or JAK1. (A) Plasmids of ANXA1, pSTAT-1/2-luc (200 ng) and pRL-TK (20 ng), were transfected into HEK293T cells. After 24 h, the cells were treated or untreated with IFN-β for 6 h, and the cell lysates were detected with the relative intensity of luciferase. EV is the negative control. (B) myc-ANXA1 and Flag-JAK1 were transfected into HEK293T cells. After 24 h, cell lysates were subjected to co-IP assay. Anti-myc antibody was used as IP primary antibody. (C and D) HEK293T cell lysates were subjected to the co-IP assay. Anti-ANXA1 antibody was used as IP primary antibody. (E) EV or myc-ANXA1 together with Flag-JAK1 were transfected into HeLa cells. After 24 h, the cells were fixed and subjected to confocal imaging. (F) EV or myc-ANXA1 together with Flag-STAT1 were transfected into HeLa cells. After 24 h, the cells were stimulated with IFN-β for 6 h, and the cells were fixed and subjected to confocal imaging. (G and H) EV or myc-ANXA1 together with Flag-STAT1 or Flag-JAK1 were transfected into HEK293T cells. After 24 h, the cells treated or untreated with IFN-β for 6 h, and the cell lysates were subjected to the co-IP assay. Anti-myc antibody was used as IP primary antibody.

To further investigate the relationship between ANXA1 and the IFN-I downstream signaling pathway, we investigated which components of the JAK-STAT signal transduction pathway are involved in ANXA1 regulation. The results of luciferase assay showed that ANXA1 promotes the activation of STAT1 and STAT2 dimer-binding promoter (STAT1/2-luc) (Fig. 6A). ANXA1 significantly increased STAT1 phosphorylation but not that of STAT2 after IFN-I stimulation (Fig. 3F). Co-IP assays were performed to detect the association of ANXA1 with JAK1 or STAT1. The results showed that ANXA1 interacts with JAK1 and STAT1 (Fig. 6B to D). Confocal microscopy results also demonstrated that ANXA1 colocalized with JAK1 and STAT1 (Fig. 6E and F). To assess the effect of IFN-β on the interaction between ANXA1 and JAK1 or STAT1, ANXA1 was cotransfected with JAK1 or STAT1 into HEK293 cells, and the cells were then stimulated with IFN-β for 6 h. As shown in Fig. 6G and H, IFN-β treatment did not affect ANXA1-JAK1 or ANXA1-STAT1 interaction. Taken together, ANXA1 interaction with JAK1 and STAT1 might promote JAK-STAT signal transduction.

### Effect of ANXA1 on FMDV replication.

**FIG 7:**
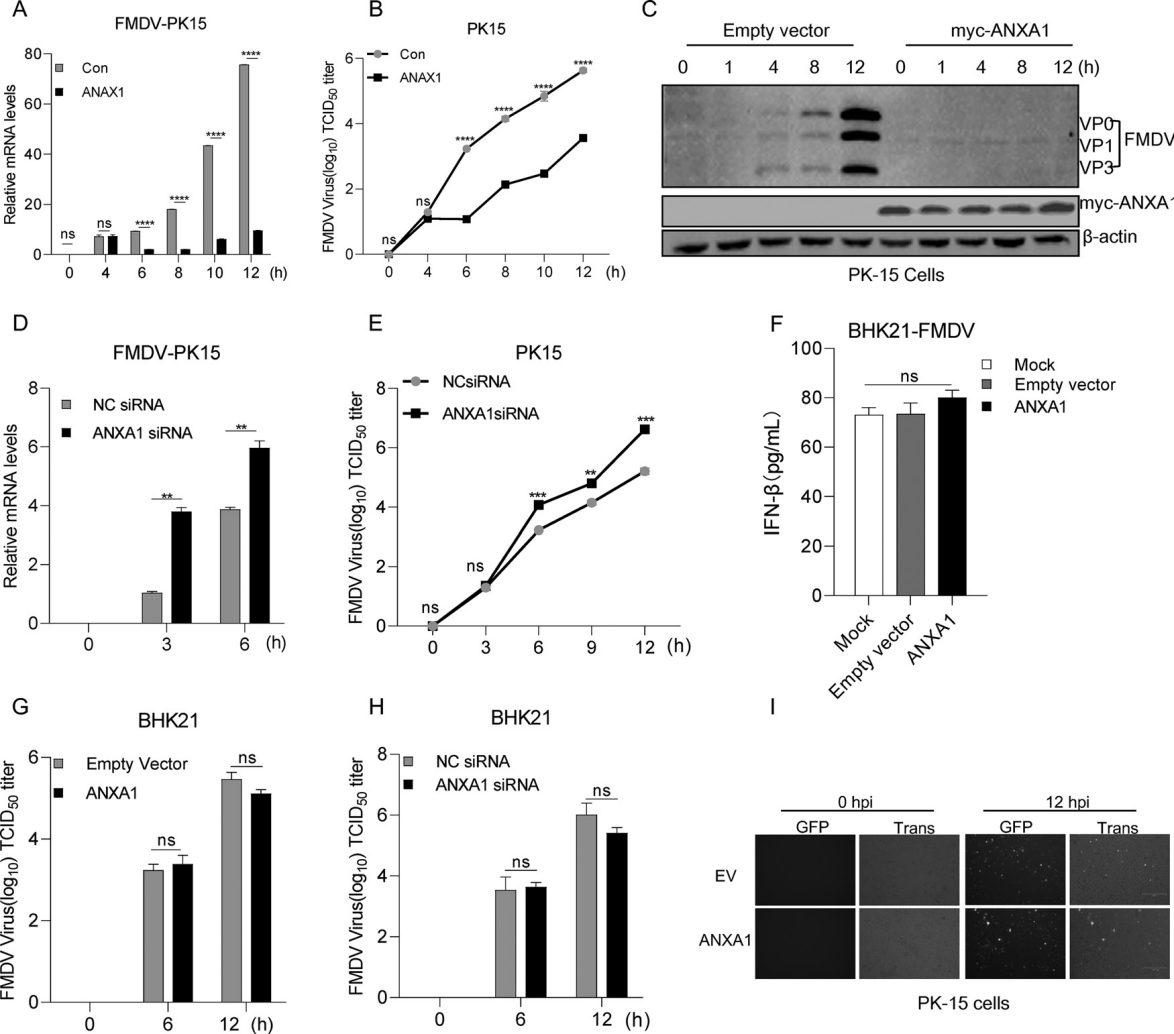
Effect of ANXA1 on FMDV replication. (A) ANXA1 or EV (pcDNA 3.1 empty vector, EV) was transfected into PK15 cells. After 24 h, the cells were infected with FMDV (MOI of 4) at the indicated time points, and cellular RNA was then extracted and reverse transcribed into cDNA. The relative mRNA level was detected by qPCR. (B) ANXA1 or EV (pcDNA 3.1) was transfected into PK15 cells. After 24 h, the cells were infected with FMDV (MOI of 4) at the indicated time points, and the FMDV supernatant was collected after freezing and thawing three times. The FMDV titers (TCID_50_) were detected with BHK21 cells. (C) ANXA1 or EV (pcDNA 3.1) was transfected into PK15 cells. After 24 h, the cells were infected with FMDV (MOI of 4) at the indicated time points, and cell lysates were then subjected to Western blotting. (D) ANXA1 siRNA or negative siRNA (NC siRNA) was transfected into PK15 cells, and after 24 h, the cells were infected with FMDV at the indicated time points. Cellular RNA then was extracted and reverse transcribed into cDNA. The relative mRNA levels were detected by qPCR. (E) ANXA1 siRNA or negative siRNA (NC siRNA) was transfected into PK15 cells, and after 24 h, the cells were infected with FMDV at the indicated time points. The FMDV supernatant was collected after freezing and thawing three times. The FMDV titers (TCID_50_) were detected with BHK21 cells. (F) ANXA1 or EV was transfected into BHK21 cells. After 24 h, the cells were infected with FMDV for 12 h; the supernatant was then collected for IFN-β ELISA detection. (G and H) EV, ANXA1, ANXA1 negative siRNA control (NC siRNA), or ANXA1 siRNA was transfected into BHK21 cells. After 24 h, the cells were infected with FMDV at indicated time points. The FMDV titers were then detected. (I) EV or ANXA1 was transfected into BHK21 cells. After 24 h, the cells were infected with FMDV for 12 h. The medium supernatant was then collected, and UV light was used to inactive the medium supernatant for 2 h. PK-15 cells were treated with the inactive supernatant for 6 h, and the cells were infected with VSV-GFP at indicated time points. Green fluorescence was observed. Data are representative of three independent experiments. The data are expressed as means ± SEM; *, *P < *0.05; **, *P < *0.01; ***, *P < *0.001; ****, *P < *0.0001 (two-way ANOVA; GraphPad Prism 8.3.0).

To assess the effect of ANXA1 on FMDV replication, we transfected ANXA1 into PK15 cells and then detected the relative mRNA levels of FMDV compared to that in the control. As shown in Fig. 7A, after ANXA1 transfection, we found that ANXA1 overexpression decreased FMDV mRNA expression compared to that in the control. The FMDV titers were determined by a 50% tissue culture infective dose (TCID_50_) assay, and we found that ANXA1 overexpression reduced FMDV titer compared to that in the control (Fig. 7B). Consistent with this, the results of Western blotting showed that the expression levels of the FMDV structural proteins VP0, VP1, and VP3 were decreased after ANXA1 transfection (Fig. 7C). Moreover, we transfected ANXA1 siRNA into PK15 cells and detected the extent of FMDV replication. The results showed that ANXA1 knockdown increased the relative mRNA level and the titer of FMDV compared to that in the control (Fig. 7D and E).

To assess whether ANXA1 inhibits FMDV replication depending on IFN-I production, we investigated the effect of ANXA1 on FMDV replication in BHK21 cells (IFN-β-deficient cells). As shown in Fig. 7F, regardless of overexpression of ANXA1, IFN-β levels did not change after FMDV infection in BHK21 cells compared to that in controls. Next, we assessed the FMDV titer in FMDV-infected BHK21 cells overexpressing ANXA1 or ANXA1 siRNA. As shown in Fig. 7G and H, after ANXA1 or ANXA1 siRNA overexpression, FMDV TCID_50_ was similar to that of the control. In addition, to determine the effect of ANXA1 on antiviral status, ANXA1 was transfected into BHK21 cells and then infected with FMDV for 12 h. The supernatant was then collected, and UV light was used to inactivate FMDV particles. PK15 cells were infected with VSV-GFP (vesicular stomatitis virus with green fluorescent protein tag) after supernatant treatment for 6 h. The results showed that in ANXA1-mediated treatment of PK15 cell supernatant, the VSV-GFP fluorescence showed no difference compared to that in EV-mediated PK15 control cells (Fig. 7I). These results suggest that ANXA1-mediated IFN-β production affects FMDV replication. Taken together, the expression of ANXA1 in IFN-β-producing cells inhibited the replication of FMDV.

### FMDV 3A inhibits ANXA1-promoted innate immune response.

**FIG 8:**
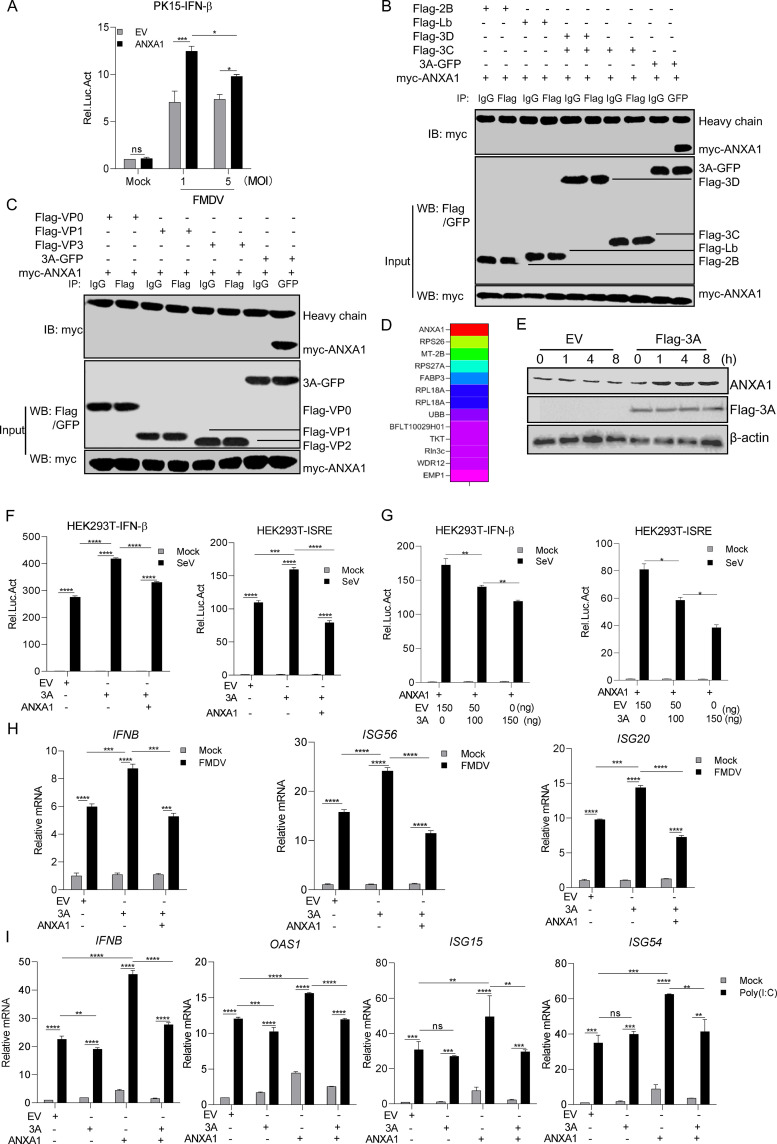
FMDV 3A protein inhibited the ANXA1-promoting function on IFN-I production. (A) ANXA1 (1 μg), pRL-TK (40 ng), and pIFN-β-luc (400 ng) plasmids were transfected into PK-15 cells. After 24 h, the cells were untreated or treated with FMDV (MOI of 1 or 5). After 12 h, the cell lysates were detected with the relative intensity of luciferase. (B and C) HEK293 cells were separately transfected with ANXA1 and FMDV-encoded proteins. After 24 h, the cell lysates were subjected to IP detection. Anti-Flag or anti-GFP antibodies were used as the IP primary antibodies. (D) FMDV-encoded 3A as a bait protein and PK15 cell-derived genes as a yeast library, using yeast two-hybrid technology; we screened that the FMDV 3A protein associated with ANXA1 and other proteins. (E) FMDV 3A plasmid (Flag-3A) or empty plasmid (EV) was transfected into PK15 cells, and after 24 h, the cell lysates were subjected to Western blotting. (F) Plasmids of EV (400 ng), ANXA1 (300 ng) plus EV (100 ng), ANXA1 (300 ng) plus 3A (100 ng), and pRL-TK (20 ng) together with pIFN-β-luc or pISRE-luc (200 ng) were transfected into HEK293T cells. After 24 h, the cells were infected with SeV, and after 12 h, the cell lysates were detected with the relative intensity of luciferase. (G) ANXA1 (100 ng) or empty plasmid (100 ng) and the indicated dose of 3A, pRL-TK (20 ng) together with pIFN-β-luc, or pISRE-luc were transfected into HEK293T cells. After 24 h, the cells were treated or untreated with SeV, and after 12 h, the cell lysates were detected with the relative intensity of luciferase. (H) Plasmids of EV (1 μg), ANXA1 (500 ng) plus EV (500 ng), or ANXA1 plus 3A (500 ng) were transfected into PK15 cells. After 24 h, the cells were infected with FMDV (MOI of 4). After 9 h, cellular RNA was extracted and reverse transcribed into cDNA. The relative mRNA levels were detected by qPCR. Data are representative of three independent experiments. (I) Plasmids of EV (1 μg), ANXA1 (500 ng) plus EV (500 ng), or ANXA1 plus 3A (500 ng) were transfected into PK-15 cells. After 24 h, the cells were transfected with poly(I:C) (1 μg). After 18 h, cellular RNA was extracted and reverse transcribed into cDNA. The relative mRNA level was detected by qPCR. Data are representative of three independent experiments. The data are expressed as means ± SEM; *, *P < *0.05; **, *P < *0.01; ***, *P < *0.001; ****, *P < *0.0001 (two-way ANOVA; GraphPad Prism 8.3.0).

Next, the effect of FMDV on ANXA1-promoted innate immune response was determined. As shown in Fig. 8A, in the luciferase reporter gene assay, ANXA1 increased FMDV-induced IFN-β activation in PK-15 cells compared to that in EV-transfected cells. In addition, compared to a lower titer of FMDV-infected cells, a higher titer of FMDV significantly inhibited IFN-β activation in ANXA1-overexpressing PK15 cells (Fig. 8A).

To determine whether FMDV proteins are involved in ANXA1 regulation, HEK293T cells were separately transfected with the FMDV protein and ANXA1. We found that FMDV 3A interacted with ANXA1 but not with others (Fig. 8B and C). To further confirm that 3A combines with ANXA1, a yeast two-hybrid screen was performed with FMDV 3A as the bait. Colonies were screened, and the positive clones were subjected to sequencing. As shown in Fig. 8D, we found the presence of ANXA1 in the yeast two-hybrid screening assay. Consistent with the results of the yeast two-hybrid screening assay, we assessed the interaction between 3A and ANXA1 with co-IP, and the results suggest that ANXA1 physically interacted with 3A (data not shown).

To determine whether FMDV 3A is involved in the regulation of ANXA1 function, in the transient-transfection experiment, ANXA1 protein expression was increased after FMDV 3A transfection (Fig. 8E). The effect of 3A on ANXA1-promoted IFN-I production was also examined. The luciferase assay demonstrated that 3A inhibited ANXA1-promoted IFN-β activation in a dose-dependent manner (Fig. 8F and G). Consistent with this, 3A also inhibited the mRNA expression of *IFNB*, *ISG56*, and *ISG20*, which was induced by FMDV after ANXA1 overexpression (Fig. 8H). Because the FMDV contains the 3A protein, we used poly(I:C) to imitate the RNA genome; as shown in Fig. 8I, 3A decreased the mRNA levels of the *ISGs*, namely, *IFNB*, *OAS1*, *ISG15*, and *ISG54*, which was induced by poly(I:C) after ANXA1 overexpression. Taken together, 3A inhibited ANXA1-promoted IFN-I production.

### FMDV 3A interacted with ANXA1 to promote FMDV replication.

**FIG 9:**
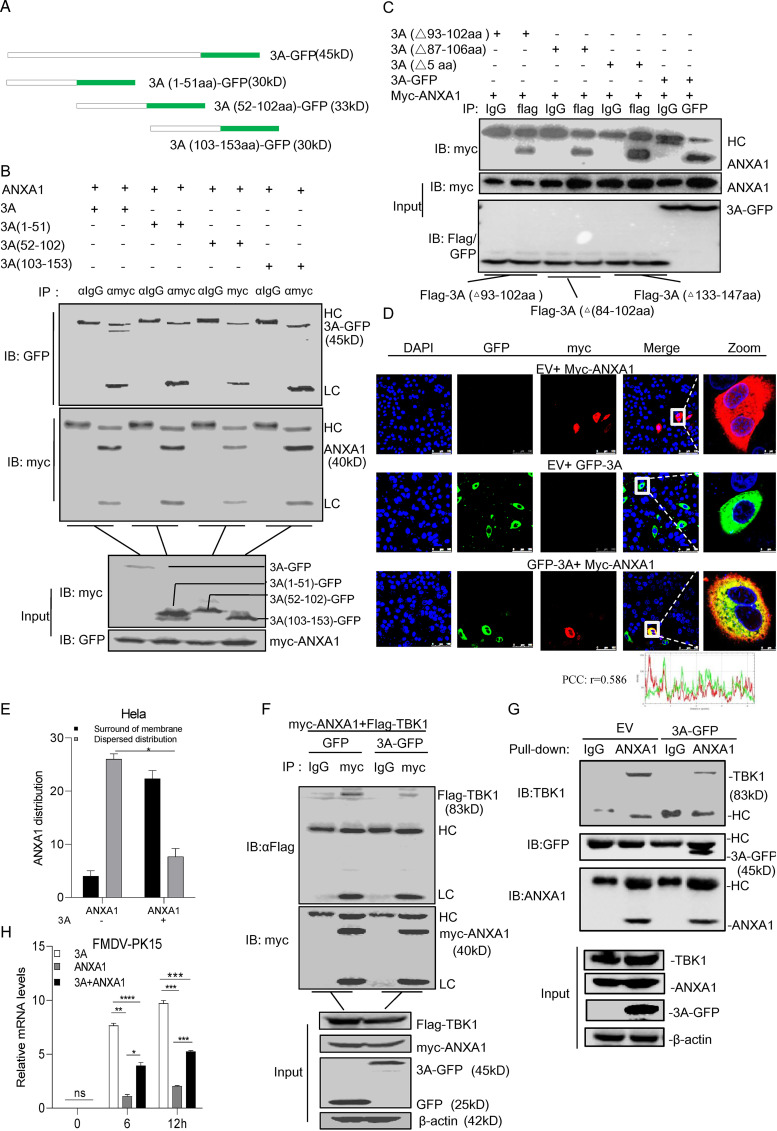
Full-length FMDV 3A protein impaired ANXA1-TBK1 complex formation. (A) Diagrammatic sketch of 3A and its mutants containing a GFP tag. (B) Plasmids of ANXA1 and full-length 3A or its mutants with GFP tag were transfected into HEK293T cells. After 24 h, the cell lysates were subjected to the co-IP assay, and anti-myc monoclonal antibody was used as the IP primary antibody. (C) Plasmids of 3A-GFP, Flag-3A (aa 93 to 102, Δ93-102aa), Flag-3A (Δ87-106aa), or Flag-3A (E78G, H80C, K84 N, A97P, and R127I of 5-aa-site mutant, Δ5aa) deletion together with myc-ANXA1 were transfected into HEK293 cells. After 24 h, cell lysates were subjected to IP detection. IgG, Flag, or GFP was used as IP primary antibody. (D and E) Plasmids of FMDV 3A-GFP or EV together with myc-ANXA1 or EV were transfected into HeLa cells. (D) After 24 h, the cells were fixed and subjected to confocal imaging, and the distribution of ANXA1 in the cells was analyzed. (E) Thirty cells were observed, and the number of cells in which ANXA1 was dispersed in the cytoplasm and nucleus and the number of cells in which ANXA1 was distributed around the membrane was statistically analyzed. (F) Plasmids of myc-ANXA1 and Flag-TBK1 and GFP or 3A-GFP were transfected into HEK293 cells. After 24 h, the cell lysates were subjected to the co-IP assay, and anti-myc monoclonal antibody was used as IP primary antibody. (G) Plasmids of His-ANXA1 or His-TBK1 were transferred into bacteria of Escherichia coli (BL-21), and the proteins were purified. EV or 3A-GFP was transfected into HEK293 cells. After 24 h, the cell lysates were collected. Next, the same concentration of EV or 3A cell lysate was added into ANXA1 and TBK1 mixture at 4°C for 4 h. The mixtures were subjected to the pulldown assay, and anti-ANXA1 monoclonal antibody was used as the pulldown primary antibody. (H) Plasmids of 3A (500 ng) plus EV (500 ng), ANXA1 (500 ng) plus EV (500 ng), or ANXA1 (500 ng) plus 3A (500 ng) were transfected into PK15 cells. After 24 h, the cells were infected with FMDV at indicated time points. Cellular RNA was extracted and reverse transcribed into cDNA. The relative mRNA level was detected by qPCR. Data are representative of three independent experiments. The data are expressed as means ± SEM; *, *P < *0.05; **, *P < *0.01; ***, *P < *0.001; ****, *P < *0.0001 (two-way ANOVA; GraphPad Prism 8.3.0).

To investigate the interaction domain of 3A that is responsible for binding to ANXA1, we constructed deletion mutants of 3A containing a GFP tag, including 3A with amino acids 1 to 51 (1-51aa)-GFP, 3A (52-102aa)-GFP, and 3A (103-153aa)-GFP (Fig. 9A). The interactions between 3A-GFP deletion mutants and ANXA1 were evaluated. As shown in Fig. 9B, the full-length 3A protein could bind to ANXA1, suggesting that ANXA1 combines with the conformational structure of 3A.

Several viruses with mutations in 3A have been identified to affect the virulence of FMDV. To determine whether these 3A mutants affect binding to ANXA1, 3A-(87-106aa deletion mutant), 3A-(93-102aa deletion mutant), or 3A-(E78G, H80C, K84 N, A97P, and R127I substitution mutant) together with ANXA1 were transfected into HEK293 cells. As shown in Fig. 9C, all the mutants bound to ANXA1, suggesting that 3A virulence-related mutants did not affect the interaction with ANXA1.

A confocal microscopy experiment was performed to demonstrate whether ANXA1 and 3A colocalize at similar subcellular positions. The results showed that 3A was distributed in the cytoplasm, while ANXA1 was expressed in the cytoplasm and nucleus. 3A and ANXA1 showed remarkable colocalization in the cytoplasm. The PCC results showed that 3A and ANXA1 have high correlation. We also found that 3A likely promotes the accumulation of ANXA1 around the cell membrane (Fig. 9D). We statistically analyzed the number of cells in which ANXA1 was distributed clustered around the membrane. We found that ANXA1 was distributed in both cytoplasm and nucleus in 87% of cells without 3A expression, while ANXA1 was clustered around the membrane in 70% of cells after 3A expression (Fig. 9E).

To reveal the mechanisms by which FMDV 3A protein inhibits ANXA1-promoted innate immune response, 3A together with ANXA1 and TBK1 were coexpressed in HEK293 cells. As shown in Fig. 9F, 3A expression inhibited ANXA1 and TBK1 complex formation. To prove the accuracy of this finding, we expressed TBK1 and ANXA1 proteins in Escherichia coli and purified these two proteins. Next, we transfected EV or 3A plasmid into HEK293 cells and collected the cell lysate. We then added the same concentration of EV or 3A cell lysate into the ANXA1 and TBK1 mixture. A pulldown assay was performed to detect the effect of 3A on the binding of ANXA1 with TBK1. Consistent with this, 3A impaired ANXA1 and TBK1 interaction (Fig. 9G). Subsequently, we separately transfected HEK293 cells with 3A, ANXA1, or 3A plus ANXA1, and we found that 3A decreased the inhibitory effect of ANXA1 on FMDV replication (Fig. 9H).

Collectively, these findings suggest that the interaction of 3A and ANXA1 is critical for FMDV replication.

## DISCUSSION

ANXA1 is a member of the annexin family (20) and is characterized as an anti-inflammatory or modulating protein because of its effect on the modulation of the immune response. It is secreted from cells to exert anti-inflammatory effects, although it is lacking a signaling peptide (21). De Oliveira Cardoso et al. reported that ANXA1 is also involved in antiparasitic effects in placentas infected with Toxoplasma gondii (22). A recent study showed that ANXA1 is associated with NEMO or RIP1, which regulates NF-κB signaling in cancer cells (23). Another study found that ANXA1 promotes TLR3- and TLR4-induced IFN-β production to influence the innate immune system (13). Yap et al. also confirmed that ANXA1 promotes RIG-I-dependent signaling and apoptosis through the regulation of the IRF3-IFNAR-STAT1-IFIT1 pathway in A549 lung epithelial cells (15). However, the effect of ANXA1 on RNA virus-induced RLR signaling remains unclear. In the present study, we first demonstrated that RNA virus infection increased ANXA1 expression, which may explain the regulatory functions of ANXA1 under RNA virus stimulation (24–26). Interestingly, we found that ANXA1 showed two bands after FMDV infection at 16 h. Full-length and truncated forms of ANXA1 are often observed in extracellular fluids and inflammatory exudates (27–29). Previous studies have shown that several proteases, such as plasmin and cathepsin D, cleave ANXA1 within the first 30 amino acids, while calpain 1 cleaves ANXA1 after lys-26 (30). FMDV 3C and L^pro^ can cleave host proteins (14, 16); however, we found that these proteins are not involved in the production of two bands of ANXA1. The exact mechanism needs to be further studied.

RIG-I or MDA5 senses cytoplasmic viral RNA to activate MAVS to recruit TBK1 and activate IRF3 for inducing IFN-I production (6). Thus, the regulators that trigger the RLR response represent a critical component in the molecular mechanisms of the antiviral innate immune response. In recent years, multiple proteins have been identified to control RLR signal transduction, thereby maintaining the balance of physiological functions; for example, GSK3β interacts with TBK1, resulting in TBK1 phosphorylation at Ser172 and facilitating TBK1 activation after viral infection (31), and SHIP-1 (inositol 5′-phosphatase) targets TBK1 to negatively regulate TLR3- or TLR4-induced IFN-β production (32). ANXA1 promotes IFN-β induction through the TLR pathway after lipopolysaccharide (LPS) stimulation. The present study showed that ANXA1 acted as a positive mediator to regulate RNA virus-induced RLR signaling. As shown in Fig. 2, we demonstrated that ANXA1 overexpression inhibits FMDV and SeV replication. Consistent with this, ANXA1 was shown to negatively regulate hepatitis C virus (HCV) infection by inhibiting viral RNA replication (33). In contrast, Arora et al. reported that ANXA1 enhanced influenza A virus infection in A549 cells, which may facilitate the process of nuclear vRNP accumulation, thereby resulting in increased viral loads (34). To investigate the effect of ANXA1 on FMDV replication, we demonstrated that ANXA1 overexpression in PK15 cells significantly decreased viral replication after FMDV infection. As shown in Fig. 7, we found that ANXA1 inhibits FMDV replication depending on IFN-I production. These studies suggest that ANXA1 plays various roles in regulating different viral infections.

Bist et al. demonstrated that ANXA1 facilitates LPS-induced IFN-β activation (13). Subsequently, we found that ANXA1 overexpression promotes RNA virus-induced IFN-β production. By using SeV or FMDV stimulation, we observed that ANXA1-deficient or ANXA1-knockdown cells showed impaired IFN-β production. We also found that RNA virus-induced ANXA1 promoted its function through the MAVS-IRF3 axis but not through the MAVS-NF-κB signaling cascade.

Yap et al. reported that ANXA1 promotes RIG-I-dependent signaling and apoptosis through the regulation of the IRF3-IFNAR-STAT1-IFIT1 pathway in A549 lung epithelial cells (15). ANXA1 does not bind to RIG-I when RIG-I is activated with 5′-ppp (15). However, the mechanism remains unclear. As shown in Fig. 5A, we found that ANXA1 activated IFN-I production through RIG-I, MDA5, and TBK1 expression; however, during VISA overexpression, IFN-I production did not change when ANXA1 was present. These findings suggest that ANXA1 promotes IFN-I production at or upstream of the level of VISA and IRF3. Here, we demonstrated that ANXA1 interacts with VISA and TBK1. We speculated that ANXA1 influences the posttranslational modification of VISA or the formation and stability of RIG-I-VISA and MDA5-VISA complexes during RLR signaling activation. The kinase TBK1 is one of the essential components of the RLR signaling pathway (6). Upstream of the IRF3 level, we found that ANXA1 interacts with TBK1 to promote IFN-I production during RLR signaling activation. ANXA1 binds with TBK1 to regulate IFN-I production after TLR3 or TLR4 activation (11). Similar to the previous report, we observed that ANXA1 interacts and colocalizes with TBK1, which increases phosphorylation of TBK1 and IRF3 after RNA viral infection. We speculated that the ANXA1 interaction with TBK1 promotes the autophosphorylation of TBK1, K63-polyubiquitination of TBK1, or TBK1-IRF3 complex formation after RNA viral infection. The present study showed that ANXA1 also interacts with JAK1 and STAT1 to affect JAK-STAT signaling pathway. These results indicate that ANXA1 regulates the area upstream of VISA and IRF3 independently in IFN-I production. We confirmed that after IFN-β stimulation, ANXA1 did not affect ANXA1-STAT1 and ANXA1-JAK1 complex formation. We speculated that ANXA1 changes the posttranslational modification of signaling proteins to promote IFN-I production and signaling transduction. The specific mechanisms, however, require further studies.

It is a contradiction that the host enhances the innate immune response with some host proteins and the virus creates a “preferred microenvironment” for virus replication. Conversely, with the coevolution of viruses and the innate immune system, pathogens developed the ability to evade or actively hinder antiviral responses from every aspect of IFN-β production. Viral proteins inhibit IFN-I production by creating a beneficial environment for virus replication. For instance, FMDV L^pro^ (leader proteinase) inhibits TBK1 ubiquitination to modulate TBK1 activity and suppress IFN-I responses (35). The FMDV 3A protein interacts with vimentin to modulate FMDV replication negatively (36). The NS3 protein of HCV interacts with TBK1 to disrupt TBK1-IRF3 complex formation (37). However, the molecular mechanisms underlying the ability of FMDV proteins to affect replication are not entirely understood. The FMDV 3A protein is a critical player in constructing the viral replication complex essential for replication. Therefore, studies on the effect of FMDV 3A will provide insights for understanding the mechanistic replication of FMDV. The present study found that FMDV 3A interacts with ANXA1 to inhibit IFN-I production and create a favorable environment for FMDV replication. A previous study reported that FMDV also used the 3A protein to suppress adverse factors, such as FMDV 3A binding to vimentin to positively modulate viral replication (36) and FMDV 3A interacting with DDX56 to reduce the phosphorylation of IRF3. Here, we demonstrated that the full-length FMDV 3A interacted and colocalized with ANXA1 to antagonize RNA virus-induced IFN-I production.

Interestingly, 3A colocalizes with ANXA1 and might promote ANXA1 to accumulate around the cell membrane. Previous studies have shown that upon neutrophil stimulation and adherence to the endothelium, ANXA1 is externalized to the plasma membrane. Because 3A anchors to intracellular membranes, 3A membrane-binding properties indicate that FMDV 3A plays a role and interacts with the membrane protein ANXA1 during FMDV replication. The 3A protein also antagonized the effect of ANXA1 on FMDV replication. Corresponding to FMDV, 3A interacts with ANXA1 to impair ANXA1 and TBK1 complex formation.

Several viruses with mutations in 3A have been identified to influence the virulence of FMDV (4, 5, 38). We assessed the effect of 3A mutants on ANXA1 interaction. The results showed that 3A mutants do not affect ANXA1 binding. This suggests that the function of 3A as a virulence factor is not related to ANXA1 regulation.

**FIG 10:**
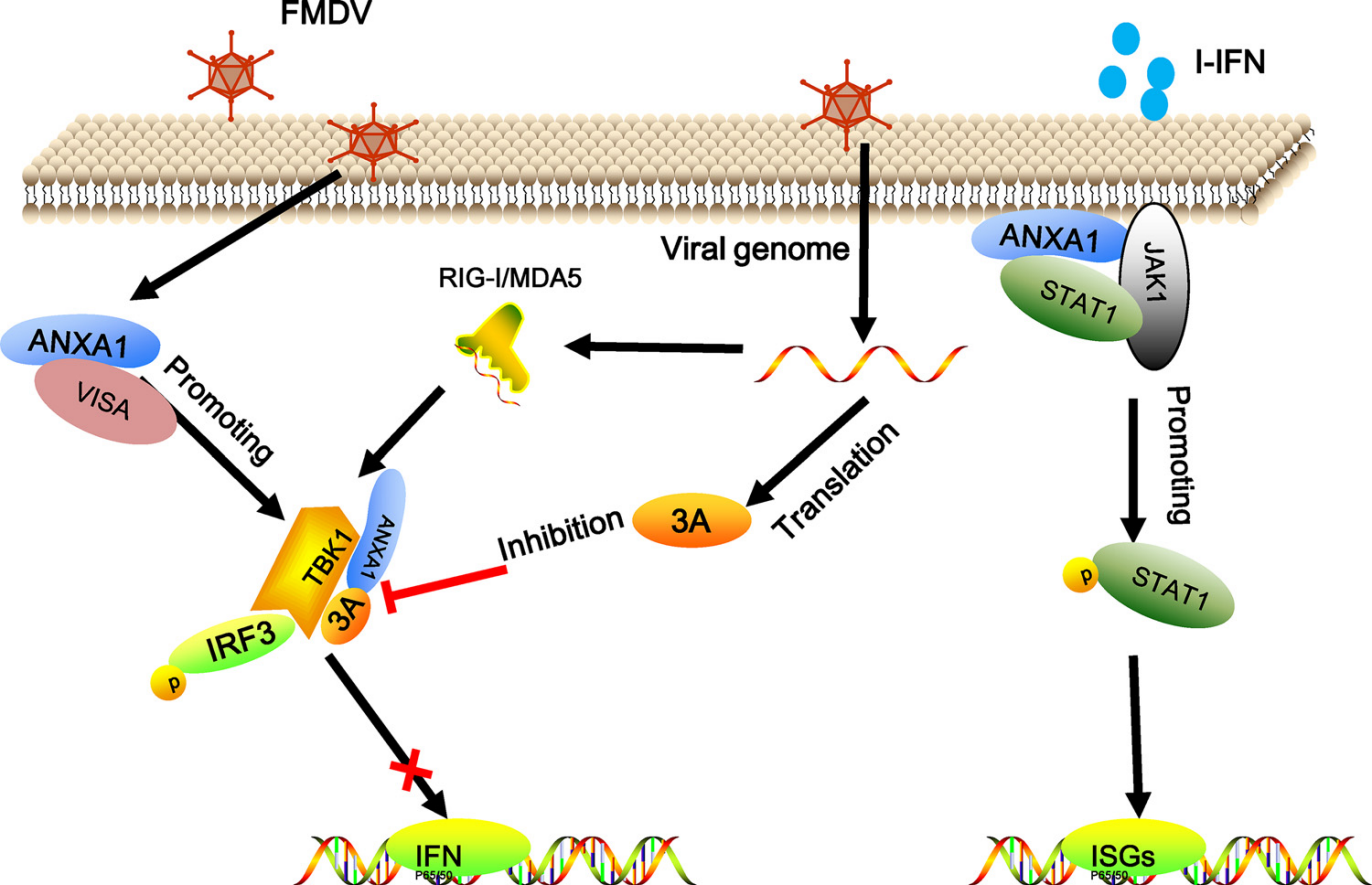
Model of FMDV 3A inhibiting the ANXA1-promoting function on IFN-I production. FMDV infection increased ANXA1 expression levels, and ANXA1 interacts with VISA and TBK1 to increase IFN-I production. ANXA1 also interacts with JAK1 and STAT1 to promote IFN-β-stimulated signaling transduction. However, the FMDV 3A protein interacted with ANXA1 to disrupt ANXA1-TBK1 complex formation, thus leading to the inhibition of IFN-I production and ultimately promote FMDV replication.

Collectively, our results showed that FMDV 3A interacts with ANXA1 to disrupt ANXA1-TBK1 complex formation to regulate IFN-I production negatively and that ANXA1 is essential for inhibiting FMDV replication (Fig. 10). The present study provides insights into the mechanism of ANXA1 in the virus-induced innate immune response and reveals that FMDV 3A antagonizes the effect of ANXA1 on viral replication. Further studies are required to provide a more thorough understanding of the role of 3A in FMDV replication.

## MATERIALS AND METHODS

### Cells, viruses, and reagents.

Human embryonic kidney 293T (HEK293T), human embryonic kidney 293 (HEK293), baby hamster kidney 21 (BHK21), HeLa (Henrietta Lacks), and porcine kidney 15 (PK15) cell lines were maintained in our laboratory, while ANXA1 knockout cells and wild-type cells were constructed by EDIGENE Biotechnology Co., Ltd. (Beijing, China). HEK293 and ANXA1 knockout HeLa cells and HeLa wild-type cells were cultured in Dulbecco's modified Eagle’s medium (DMEM; Gibco), and BHK21 and PK15 cells were cultured in modified Eagle’s medium (MEM; Gibco) supplemented with 10% or 20% fetal bovine serum (FBS), 100 U/mL penicillin, and 100 μg/mL streptomycin at 37°C with 5% CO_2_.

Herpes simplex virus (HSV), Sendai virus (SeV), and wild-type FMDV (FMDV/O/BY/CHA/2010) were stored in our laboratory. HSV and SeV were propagated in embryonated chicken eggs. FMDV replication and titration were performed in BHK21 cells. Primary antibodies against Flag, hemagglutinin (HA), and myc were purchased from Sigma. Antibodies against ANXA1, TBK1, β-actin, IRF3, p-IRF3, and p-TBK1 were obtained from Cell Signaling Technology (CST). The antibodies used in the present study were specific for both human- and pig-derived proteins. Lipofectamine 2000 and Lipofectamine 3000 were purchased from Invivogene Biotech Co., Ltd., and TRIzol was purchased from Invitrogen ((Thermo Fisher Scientific [China] Co., Ltd., China).

### Plasmids and transfection.

The cDNA fragment of 3A was amplified from total RNA extracted from FMDV/O/BY/CHA/2010. The DNA fragments of ANXA1, VISA, STAT1, JAK1, and TBK1 were generated by PCR amplification of the total RNA of PK15 cells. Expression plasmids were constructed by cloning the indicated sequences into the plasmid pcDNA3.1. Lipofectamine 2000 or 3000 (Invitrogen) was used for transfection according to the manufacturer’s instructions.

### RNA isolation and quantitative PCR assay.

RNA was extracted from HEK293T, PK15, or HeLa cells by using an RNA extraction kit, and cDNA was synthesized using the MLV-reverse transcription system (Promega) according to the manufacturer’s instructions. SYBR green real-time PCR was used to analyze gene expression. Data were normalized to the β-actin expression level. Real-time quantitative PCR (qPCR) primers are listed in Table 1.

**TABLE 1:** Primers used for qPCR

Primer^*a*^	Sequence
pIFN-β forward	5′TGCAACCACCACAATTCC3′
pIFN-β reverse	5′CTGAGAATGCCGAAGATCTG3′
pANXA1 forward	5′TTGGAAGAAGGCAGGGAAAA3′
pANXA1 reverse	5′TGAGGATGGATTGAAGGTAGGATAG3′
pISG56 forward	5′ACGTAACTGAAAATCCACAAGA3′
pISG56 reverse	5′TGCTCCAGACTATCCTTGACCT3′
pISG20 forward	5′CTCCTGCACAAGAGCATCCA3′
pISG20 reverse	5′CATCGTTGCCTTCGCATCT3′
pGAPDH forward	5′ACATGGCCTCCAAGGAGTAAGA3′
pGAPDH reverse	5′GATCGAGTTGGGGCTGTGACT3′
FMDV forward	5′ACTGGGTTTTACAAACCTGTGA3′
FMDV reverse	5′GCGAGTCCTGCCACGGA3′
hIFN-β forward	5′TTGTTGAGAACCTCCTGGCT3′
hIFN-β reverse	5′TGACTATGGTCCAGGCACAG3′
hANXA1 forward	5′GGATCCATGGCAATGGTATCAGA3′
hANXA1 reverse	5′CTCGAGGTTTAGTTTCCTCCACA3′
hISG56 Forward	5′GCCTTGCTGAAGTGTGGAGGAA3′
hISG56 reverse	5′ATCCAGGCGATAGGCAGAGATC3′
hISG54 forward	5′CACCTCTGGACTGGCAATAGC3′
hISG54 Reverse	5′GTCAGGATTCAGCCGAATGG3′
hGAPDH forward	5′GAGTCAACGGATTTGGTCGT3′
hGAPDH reverse	5′GACAAGCTTCCCGTTCTCAG3′
SeV forward	5′TGTTATCGGATTCCTCGACGCAGTC3′
SeV reverse	5′TACTCTCCTCACCTGATCGATTATC3′
HSV-1 forward	5′TCGGCGTGGAAGAAACGAGAGA3′
HSV-1 reverse	5′CGAACGCACCCAAATCGACA3′

a p, porcine; h, human.

### Luciferase reporter assay.

HEK293T cells were cultured in a 24-well plate, and after 24 h, IFN-β-Luc or ISRE-Luc and the pRL-TK plasmid (kindly provided by HongBing Shu from Wuhan University, China) were transfected into HEK293T cells (1 × 10^5^ cells/well) along with plasmids encoding the indicated genes. The cells were further cultured for 24 h posttransfection and harvested 12 h later, and luciferase activity was determined using a dual-luciferase reporter assay system (Promega) according to the manufacturer’s protocol.

### Coimmunoprecipitation.

After transfection and stimulation, HEK293T cells were harvested and lysed with a lysis buffer for 30 min at 4°C. Cell lysates were centrifuged at 12,000 rpm for 10 min, and the supernatant (1 μg total protein) was incubated with the indicated antibodies (1 μg) and protein A/G-Sepharose beads (Roche) for 2 h at 4°C. The beads were then washed 5 times with the lysis buffer. The beads with a loading buffer were boiled for 10 min at 100°C and subjected to SDS-PAGE.

### Western blotting.

Protein samples were subjected to SDS-PAGE and transferred onto a polyvinylidene difluoride (PVDF) membrane (Millipore). The membranes were blocked with 5% nonfat milk for 1 h at room temperature. Subsequently, the membranes were incubated with specific primary antibodies for 2 h at 4°C, followed by incubation with horseradish peroxidase (HRP)-labeled secondary antibodies for 1 h at room temperature. The membrane was then subjected to ECL assay.

### Confocal microscopy.

The indicated plasmids were transfected into HEK293 cells for 24 h, washed with phosphate-buffered saline (PBS) 3 times at room temperature, fixed with 4% paraformaldehyde solution for 30 min, washed with PBS for 3 times, and permeabilized with PBS containing 1% Triton X-100 and 10% sheep serum for 30 min. After washing with PBS 3 times, the cells were blocked with PBS containing 5% bovine serum albumin and 0.1% Tween 20 for 1 h, washed 3 times with PBS, incubated with target primary antibodies for 4 h at 4°C, washed 3 times with PBS, and then incubated for 1 h with fluorescence-labeled secondary antibodies. 4′,6-Diamidino-2-phenylindole (DAPI) (Sigma) was used for nuclear staining for 30 min at room temperature. Images were acquired using a Zeiss LSM 710 confocal microscope.

### Statistic analysis.

Statistical analyses were performed using GraphPad Prism software (version 8). All data were expressed as means ± standard errors of the means (SEM). A *P* value of <0.05 was considered significant (*, *P < *0.05; **, *P < *0.01; ***, *P < *0.001; ****, *P < *0.0001).

## References

[1] Belsham GJ. 1993. Distinctive features of foot-and-mouth disease virus, a member of the picornavirus family; aspects of virus protein synthesis, protein processing and structure. Prog Biophys Mol Biol 60:241–260. 10.1016/0079-6107(93)90016-d. 8396787 PMC7173301

[2] Lotufo CM, Wilda M, Giraldez AN, Grigera PR, Mattion NM. 2018. Relevance of the N-terminal and major hydrophobic domains of non-structural protein 3A in the replicative process of a DNA-launched foot-and-mouth disease virus replicon. Arch Virol 163:1769–1778. 10.1007/s00705-018-3795-9. 29536193

[3] Beard CW, Mason PW. 2000. Genetic determinants of altered virulence of Taiwanese foot-and-mouth disease virus. J Virol 74:987–991. 10.1128/jvi.74.2.987-991.2000. 10623761 PMC111619

[4] Pacheco JM, Gladue DP, Holinka LG, Arzt J, Bishop E, Smoliga G, Pauszek SJ, Bracht AJ, O′Donnell V, Fernandez-Sainz I, Fletcher P, Piccone ME, Rodriguez LL, Borca MV. 2013. A partial deletion in non-structural protein 3A can attenuate foot-and-mouth disease virus in cattle. Virology 446:260–267. 10.1016/j.virol.2013.08.003. 24074589

[5] Gao H, Wang J, Zhao G, Zhu M, He Y, Xin A. 2020. Substitution 3A protein of foot-and-mouth disease virus of attenuated ZB strain rescued the viral replication and infection in bovine cells. Res Vet Sci 128:145–152. 10.1016/j.rvsc.2019.11.001. 31791012

[6] Yoneyama M, Onomoto K, Jogi M, Akaboshi T, Fujita T. 2015. Viral RNA detection by RIG-I-like receptors. Curr Opin Immunol 32:48–53. 10.1016/j.coi.2014.12.012. 25594890

[7] Mazewski C, Perez RE, Fish EN, Platanias LC. 2020. Type I interferon (IFN)-regulated activation of canonical and non-canonical signaling pathways. Front Immunol 11:606456. 10.3389/fimmu.2020.606456. 33329603 PMC7719805

[8] Castanier C, Zemirli N, Portier A, Garcin D, Bidere N, Vazquez A, Arnoult D. 2012. MAVS ubiquitination by the E3 ligase TRIM25 and degradation by the proteasome is involved in type I interferon production after activation of the antiviral RIG-I-like receptors. BMC Biol 10:44. 10.1186/1741-7007-10-44. 22626058 PMC3372421

[9] Dai T, Wu L, Wang S, Wang J, Xie F, Zhang Z, Fang X, Li J, Fang P, Li F, Jin K, Dai J, Yang B, Zhou F, van Dam H, Cai D, Huang H, Zhang L. 2018. FAF1 regulates antiviral immunity by inhibiting MAVS but is antagonized by phosphorylation upon viral infection. Cell Host Microbe 24:776–790. 10.1016/j.chom.2018.10.006. 30472208

[10] Schloer S, Hubel N, Masemann D, Pajonczyk D, Brunotte L, Ehrhardt C, Brandenburg LO, Ludwig S, Gerke V, Rescher U. 2019. The annexin A1/FPR2 signaling axis expands alveolar macrophages, limits viral replication, and attenuates pathogenesis in the murine influenza A virus infection model. FASEB J 33:12188–12199. 10.1096/fj.201901265R. 31398292 PMC6902725

[11] Ampomah PB, Kong WT, Zharkova O, Chua S, Perumal Samy R, Lim LHK. 2018. Annexins in influenza virus replication and pathogenesis. Front Pharmacol 9:1282. 10.3389/fphar.2018.01282. 30498445 PMC6249340

[12] Huggins A, Paschalidis N, Flower RJ, Perretti M, D′Acquisto F. 2009. Annexin-1-deficient dendritic cells acquire a mature phenotype during differentiation. FASEB J 23:985–996. 10.1096/fj.08-119040. 19029200

[13] Bist P, Shu S, Lee H, Arora S, Nair S, Lim JY, Dayalan J, Gasser S, Biswas SK, Fairhurst AM, Lim LH. 2013. Annexin-A1 regulates TLR-mediated IFN-beta production through an interaction with TANK-binding kinase 1. J Immunol 191:4375–4382. 10.4049/jimmunol.1301504. 24048896

[14] Liu Y, Zhu Z, Zhang M, Zheng H. 2015. Multifunctional roles of leader protein of foot-and-mouth disease viruses in suppressing host antiviral responses. Vet Res 46:127. 10.1186/s13567-015-0273-1. 26511922 PMC4625562

[15] Yap GLR, Sachaphibulkij K, Foo SL, Cui J, Fairhurst AM, Lim LHK. 2020. Annexin-A1 promotes RIG-I-dependent signaling and apoptosis via regulation of the IRF3-IFNAR-STAT1-IFIT1 pathway in A549 lung epithelial cells. Cell Death Dis 11:463. 10.1038/s41419-020-2625-7. 32541772 PMC7295754

[16] Curry S, Roque-Rosell N, Zunszain PA, Leatherbarrow RJ. 2007. Foot-and-mouth disease virus 3C protease: recent structural and functional insights into an antiviral target. Int J Biochem Cell Biol 39:1–6. 10.1016/j.biocel.2006.07.006. 16979372 PMC7185863

[17] Qu H, Li J, Yang L, Sun L, Liu W, He H. 2016. Influenza A virus-induced expression of ISG20 inhibits viral replication by interacting with nucleoprotein. Virus Genes 52:759–767. 10.1007/s11262-016-1366-2. 27342813

[18] Feng J, Wickenhagen A, Turnbull ML, Rezelj VV, Kreher F, Tilston-Lunel NL, Slack GS, Brennan B, Koudriakova E, Shaw AE, Rihn SJ, Rice CM, Bieniasz PD, Elliott RM, Shi X, Wilson SJ. 2018. Interferon-stimulated gene (ISG)-expression screening reveals the specific antibunyaviral activity of ISG20. J Virol 92:e02140-17. 10.1128/JVI.02140-17. 29695422 PMC6002717

[19] Li Y, Li C, Xue P, Zhong B, Mao AP, Ran Y, Chen H, Wang YY, Yang F, Shu HB. 2009. ISG56 is a negative-feedback regulator of virus-triggered signaling and cellular antiviral response. Proc Natl Acad Sci USA 106:7945–7950. 10.1073/pnas.0900818106. 19416887 PMC2683125

[20] Gerke V, Moss SE. 2002. Annexins: from structure to function. Physiol Rev 82:331–371. 10.1152/physrev.00030.2001. 11917092

[21] Perretti M, Dalli J. 2009. Exploiting the annexin A1 pathway for the development of novel anti-inflammatory therapeutics. Br J Pharmacol 158:936–946. 10.1111/j.1476-5381.2009.00483.x. 19845684 PMC2785517

[22] de Oliveira Cardoso MF, Moreli JB, Gomes AO, de Freitas Zanon C, Silva AE, Paulesu LR, Ietta F, Mineo JR, Ferro EA, Oliani SM. 2018. Annexin A1 peptide is able to induce an anti-parasitic effect in human placental explants infected by Toxoplasma gondii. Microb Pathog 123:153–161. 10.1016/j.micpath.2018.07.005. 30003946

[23] Bist P, Leow SC, Phua QH, Shu S, Zhuang Q, Loh WT, Nguyen TH, Zhou JB, Hooi SC, Lim LH. 2011. Annexin-1 interacts with NEMO and RIP1 to constitutively activate IKK complex and NF-kappaB: implication in breast cancer metastasis. Oncogene 30:3174–3185. 10.1038/onc.2011.28. 21383699

[24] Zhao B, Wang J, Liu L, Li X, Liu S, Xia Q, Shi J. 2016. Annexin A1 translocates to nucleus and promotes the expression of pro-inflammatory cytokines in a PKC-dependent manner after OGD/R. Sci Rep 6:27028. 10.1038/srep27028. 27426034 PMC4947919

[25] Solito E, Christian HC, Festa M, Mulla A, Tierney T, Flower RJ, Buckingham JC. 2006. Post-translational modification plays an essential role in the translocation of annexin A1 from the cytoplasm to the cell surface. FASEB J 20:1498–1500. 10.1096/fj.05-5319fje. 16720734 PMC2049060

[26] D′Acunto CW, Gbelcova H, Festa M, Ruml T. 2014. The complex understanding of annexin A1 phosphorylation. Cell Signal 26:173–178. 10.1016/j.cellsig.2013.09.020. 24103589

[27] Chung YW, Oh HY, Kim JY, Kim JH, Kim IY. 2004. Allergen-induced proteolytic cleavage of annexin-1 and activation of cytosolic phospholipase A2 in the lungs of a mouse model of asthma. Proteomics 4:3328–3334. 10.1002/pmic.200400895. 15378764

[28] Vishwanatha JK, Davis RG, Rubinstein I, Floreani A. 1998. Annexin I degradation in bronchoalveolar lavage fluids from healthy smokers: a possible mechanism of inflammation. Clin Cancer Res 4:2559–2564. 9796991

[29] Tsao FH, Meyer KC, Chen X, Rosenthal NS, Hu J. 1998. Degradation of annexin I in bronchoalveolar lavage fluid from patients with cystic fibrosis. Am J Respir Cell Mol Biol 18:120–128. 10.1165/ajrcmb.18.1.2808. 9448053

[30] Wang W, Creutz CE. 1994. Role of the amino-terminal domain in regulating interactions of annexin I with membranes: effects of amino-terminal truncation and mutagenesis of the phosphorylation sites. Biochemistry 33:275–282. 10.1021/bi00167a036. 8286349

[31] Lei CQ, Zhong B, Zhang Y, Zhang J, Wang S, Shu HB. 2010. Glycogen synthase kinase 3beta regulates IRF3 transcription factor-mediated antiviral response via activation of the kinase TBK1. Immunity 33:878–889. 10.1016/j.immuni.2010.11.021. 21145761

[32] Gabhann JN, Higgs R, Brennan K, Thomas W, Damen JE, Ben Larbi N, Krystal G, Jefferies CA. 2010. Absence of SHIP-1 results in constitutive phosphorylation of tank-binding kinase 1 and enhanced TLR3-dependent IFN-beta production. J Immunol 184:2314–2320. 10.4049/jimmunol.0902589. 20100929

[33] Hiramoto H, Dansako H, Takeda M, Satoh S, Wakita T, Ikeda M, Kato N. 2015. Annexin A1 negatively regulates viral RNA replication of hepatitis C virus. Acta Med Okayama 69:71–78. 10.18926/AMO/53335. 25899628

[34] Arora S, Lim W, Bist P, Perumalsamy R, Lukman HM, Li F, Welker LB, Yan B, Sethi G, Tambyah PA, Fairhurst AM, Alonso S, Lim LH. 2016. Influenza A virus enhances its propagation through the modulation of annexin-A1 dependent endosomal trafficking and apoptosis. Cell Death Differ 23:1243–1256. 10.1038/cdd.2016.19. 26943321 PMC4946891

[35] Wang D, Fang L, Li P, Sun L, Fan J, Zhang Q, Luo R, Liu X, Li K, Chen H, Chen Z, Xiao S. 2011. The leader proteinase of foot-and-mouth disease virus negatively regulates the type I interferon pathway by acting as a viral deubiquitinase. J Virol 85:3758–3766. 10.1128/JVI.02589-10. 21307201 PMC3126127

[36] Ma X, Ling Y, Li P, Sun P, Cao Y, Bai X, Li K, Fu Y, Zhang J, Li D, Bao H, Chen Y, Li Z, Wang Y, Lu Z, Liu Z. 2020. Cellular vimentin interacts with foot-and-mouth disease virus nonstructural protein 3A and negatively modulates viral replication. J Virol 94:e00273-20. 10.1128/JVI.00273-20. 32493819 PMC7394891

[37] Otsuka M, Kato N, Moriyama M, Taniguchi H, Wang Y, Dharel N, Kawabe T, Omata M. 2005. Interaction between the HCV NS3 protein and the host TBK1 protein leads to inhibition of cellular antiviral responses. Hepatology 41:1004–1012. 10.1002/hep.20666. 15841462

[38] O′Donnell VK, Pacheco JM, Henry TM, Mason PW. 2001. Subcellular distribution of the foot-and-mouth disease virus 3A protein in cells infected with viruses encoding wild-type and bovine-attenuated forms of 3A. Virology 287:151–162. 10.1006/viro.2001.1035. 11504550

